# Regional disparities in road traffic injuries and their determinants in Brazil, 2013

**DOI:** 10.1186/s12939-016-0433-6

**Published:** 2016-11-17

**Authors:** Otaliba Libanio Morais Neto, Ana Lúcia Andrade, Rafael Alves Guimarães, Polyana Maria Pimenta Mandacarú, Gabriela Camargo Tobias

**Affiliations:** 1 Departamento de Saúde Coletiva. Instituto de Patologia Tropical e Saúde Pública, Universidade Federal de Goiás, Rua 235, S/N, Setor Universitário, Goiânia, Goiás Cep: 74605-050 Brazil; 2 Mestrado do Programa de Pós-Graduação em Enfermagem, Universidade Federal de Goiás, Rua 227 Qd 68, S/N - Setor Leste Universitário, Goiânia, Goiás CEP: 74605-080 Brazil; 3 Centro de Excelência em Ensino, Pesquisa e Projetos – Leide das Neves Ferreira, Rua 26, 521 - Jardim Santo Antônio, Goiânia, GO 74853-070 Brazil; 4 Programa de Pós-Graduação em Medicina Tropical e Saúde Pública, Instituto de Patologia Tropical e Saúde Pública, Universidade Federal de Goiás, Rua 235, S/N, Setor Universitário, Goiânia, Goiás Cep: 74605-050 Brazil; 5 Secretaria Municipal de Saúde de Senador Canedo, Av. Dom Manoel - Res. Boa Vista, Sen. Canedo, GO 75250-000 Brazil

**Keywords:** Traffic accidents, Epidemiologic determinants, Health disparities

## Abstract

**Background:**

In recent decades middle-income countries have experienced a rapid increase in the number of cars and motorcycles. Increased deaths and hospitalizations due to road traffic injuries (RTI) has been observed in several countries as a result. In this study we assessed the determinants of RTIs in Brazil by mode of transportation and compared differences in RTI rates among macro-regions.

**Methods:**

We used data from the National Health Survey (NHS) conducted in 2013 by the Brazilian Institute of Geography and Statistics and the Ministry of Health. NHS is a comprehensive household survey which includes a representative sample (N = 60,198) of individuals aged 18 years or older. The prevalence and determinants of RTI were estimated according to different modes of transport (car/van, motorcycle, and other) and regions of the country. Bivariate and multivariable logistic regression models were applied to assess crude and adjusted odds ratios, respectively, and their 95 % CI for RTI determinants.

**Results:**

The prevalence of RTI for the Southeast, South, Central-West, Northeast and North regions of Brazil was 2.4 %, 2.9 %, 4.4 %, 3.4 % and 4.8 %, respectively, pointing to important differences among regions. High percentages of motorcyclists were observed in the Northeast and North regions. For motorcyclists, factors associated with RTIs were being male (OR = 2.6;95 % CI:2.3;3.0), aged 18–29 (OR = 3.2; 95 % CI:2.7;3.8) and 30–39 years (OR = 2.0;95 % CI:1.7;2.5), black (OR = 1.4;95 % CI:1.1;1.7), having elementary educational (OR = 1.5;95 % CI:1.1;1.9), reporting binge drinking behavior (OR = 1.3;95 % CI:1.1;1.5), and living in the Central-West (OR = 2.0;95 % CI:1.6;2.5), Northeast (OR = 1.8;95 % CI:1.5;2.1) and North (OR = 2.0;95 % CI:1.6; 2.5) regions of the country. The independent variables associated with RTI for car/van occupants were being male (OR = 1.7;95 % CI:1.4;2.1), aged 18–29 (OR = 1.5;95 % CI:1.1;2.0) and 30–39 years (OR = 2.5;95 % CI:1.9;3.2), reporting binge drinking behavior (OR = 2.0;95 % CI:1.6;2.5) and living in the South region (OR = 1.6;95 % CI:1.3;2.1).

**Conclusions:**

There were considerable regional disparities in RTI rates across Brazil’s regions. Motorcyclists contributed to the high RTI rates in these regions as did demographic factors and behaviors such as alcohol use. These findings can help guide interventions to reduce the burden of RTIs in Brazil.

## Background

Estimates by the World Bank and the Institute of Health Metrics and Evaluation show that in 2012 road traffic injuries (RTI) were the direct cause of 1.33 million deaths, and a contributing factor in a further 184,000 deaths. In the last two decades, deaths as a result of RTI have increased by 46 %, leading to an annual global loss of nearly 80 million healthy years of life [1]. The burden of RTI has significantly increased in countries that have experienced rapid economic growth (e.g., the BRICS group — Brazil, Russia, India, China and South Africa), with such countries prioritizing investment in the construction of road infrastructure and promoting industrialization with increased vehicle production and purchases, particularly cars and motorcycles [1, 2].

In recent decades in Brazil there has been an increase in the motorization rates of cars and motorcycles as a consequence of rapid economic growth. This was accompanied by an increase in the average income of Brazil’s poorest population, rapid urbanization, and economic measures to encourage the production of cars and motorcycles. Motorcycle production (1620 %) and sales (1356 %) of motorcycles sharply increased between 1990 and 2011 [3]. The Northeast region of Brazil presented the highest percentage of motorcycle sales in Brazil in 2013. In 2008, motorcycle sales in Brazil ranked fourth in the world [4]. Car production also increased by 315 % during that period [5]. These factors have shifted the mobility pattern of Brazil’s low and middle-income populations from public to private transport [6]. The effects of this rapid increase in motorization in Brazil include increased traffic congestion, environmental pollution, and injuries and deaths due to road traffic crashes [7–9].

Regarding RTI mortality in Brazil, two trends have been observed in recent decades: a short period of reduction between 1998 and 2000, involving all modes of transport, and an upward trend from 2003–2012, characterized by a reduction in pedestrian deaths but increased deaths among vehicle occupants, especially among motorcyclists [8, 10]. The main determinant of the reduction in mortality rates from 1998–2003 was the implementation of the Brazilian Traffic Code in 1998 [8, 11]. The likely contributors to the upward trend in mortality rates were increased household income and the rapid increase in motorization rates of cars and motorcycles discussed above [9, 12]. Regarding non-fatal RTIs, a 55 % increase in emergency department visits was observed between 2003 and 2013, accompanied by a 205 % increase in hospitalizations among motorcyclists [7, 13]. A number of other legislative initiatives have also been recently introduced including the ‘dry law’ in 2008, which reduced legal blood alcohol levels among drivers from 0.06 g/L to 0.02 g/L. A corresponding reduction in RTI and fatalities was observed for a short period of time between 2008 and 2009 [14].

Changes in the spatial pattern of RTI have also occurred, with a reduction in mortality rates in the Southeast region and an increase in the North and Northeast regions of the country, mainly due to increased deaths among motorcycle riders [7]. Hence, clusters of high mortality rates due to RTI were observed in the Northeast, North, and Central-West regions, especially for motorcyclists [8].

Although several studies have provided evidence on the causes of hospitalization and deaths, few Brazilian studies have analyzed the magnitude of RTI at the population level. Two surveys have recently been conducted in Brazil: the National Household Sample Survey (PNAD) in 2008 [15], and the National Health Survey (NHS) in 2013 [16]. Results from PNAD estimated a RTI prevalence of 2.5–3.5 % for males and 1.5 % for females. Individuals aged 18–24 and 25–34 years, and those with a high level of education presented the highest RTI prevalence’s. Differences in RTI among the five macro-regions of the country were also found, with the highest prevalence in the Central-West (3.3 %), followed by the South (3.0 %), Southeast (2.5 %), North (2.4 %), and Northeast (1.9 %) [15, 17].

Population surveys of RTIs in developing countries, such as Mexico and Thailand, show a lower (Mexico) and higher (Thailand) prevalence of RTI when compared with data from Brazil for the period 2008–2013. In Mexico, the prevalence of RTI in 2006 was 1.0 %, higher in males (1.3 %) and in individuals aged 20–44 years (1.3 %) [18]. In 2012, the prevalence of RTI in Mexico was 1.2 %, with higher portion of the RTIs (53 %) among occupants of vehicles with four wheels or more, followed by motorcyclists (23 %), pedestrians (13 %), and cyclists (12 %) [19]. In Thailand, a country with high RTI mortality rates [20], the prevalence of RTI in 2009 was 10 %, with motorcycles involved in 74 % of all RTIs [21]. In Hyderabad, India, the age–sex-adjusted rate for non-fatal RTIs was 20.7 %. High rates were observed in the 15–19 age group. The rate was similar for pedestrians and motorcyclists [22].

An analysis of the determinants of RTI by mode of transport in developing countries showed the following results: for motorcyclists the determinants were being male or a young male, not married, with a middle or high income, recent migration from one city to another, driving under the influence of alcohol, inexperience with driving a motorcycle, and conspicuity [21, 23, 24]; for car drivers the main determinants were being male, a young male, fatigue, aged 40–49 years, being separated, divorced, or widowed, [21] driving under the influence of alcohol, and just driving at night [23, 25].

The 2013 NHS collected data on RTIs, but to date, there has been no report of potential determinants of RTI using this dataset. We therefore analyzed RTI data from the 2013 NHS to assess RTI determinants in Brazil by mode of transport, as well as disparities in RTI rates among Brazilian macro-regions.

## Methods

### Data

In this study we used data from the NHS conducted in 2013 by the Brazilian Institute of Geography and Statistics and the Ministry of Health. The NHS is a comprehensive household survey, which included a representative sample (N = 60,198) of individuals aged 18 years or older. Participants were interviewed during household visits. Data collection was performed by trained professionals using Personal Digital Assistants. The prevalence and determinants of RTI in Brazil were estimated according to different modes of transport (car/van, motorcycle, and other) and regions of the country. Brazil consists of 27 states and a Federal District. The Federal units are grouped into five regions: North, Northeast, Southeast, South and Central-West.

### Sampling

The 2013 PNS sampling strategy consisted of a complex sample performed in three stages: (i) primary sampling unit: census tracts or set of tracts; (ii) secondary sampling unit: households; and (iii) individuals aged 18 and older. Within each stage, participants were selected using simple random sampling. The probability of selecting each individual aged 18 years and older within a household was weighted by household, adjusted by non-response rate, sex, and age calibration by the total population. Details of the sampling design and sample size are available from previous reports [10, 26, 27].

## Analytic approach

### Outcome definition

The NHS questionnaire asked participants whether they had been involved in a road traffic crash that had resulted in a non-fatal injury in the last 12 months; if so, how many crashes had they experienced in that period, what was the mode of transport they used, and whether they were a pedestrian, driver, or passenger at the time of the most serious episode.

The present study focused on two outcomes: (i) individuals who suffered an injury from a road traffic crash in the past 12 months (regardless of the number of crashes). This outcome was used to estimate RTI prevalence by region, assess victim characteristics, and enable comparison with results from 2008 PNAD; (ii) individuals who were injured in one road traffic crash only within the last 12 months. This outcome was used to estimate the prevalence and differences of RTI determinants by mode of transport. The exclusion of individuals who reported more than one RTI episode was necessary as the mode of transport was determined based on the occurrence of the most serious episode over the survey reference period. For this outcome, three categories were considered: (i) motorcycle driver or passenger; (ii) car/van driver or passenger; and (iii) other (including pedestrian, cyclist, bus driver or passenger, truck driver or passenger, and occupant of other modes of transport). The ‘other’ modes of transport were pooled together because of their small frequency.

Determinants for this investigation were: (i) sex (male and female) as a proxy for gender [28, 29]; (ii) age groups 18–29 years (young adults), 30–39 years and 40–59 years (adults), and 60 years and older (older adults); (iii) race/skin color (white, brown, and black -- Asian and indigenous categories were too small to be considered in this analysis) as a proxy for ethnic identity and socioeconomic level [29, 30]; (iv) educational level (college or higher, high school, elementary school and lower than elementary school/illiterate) as a proxy for socioeconomic level [24, 31]; (v) living with a husband/wife or partner (yes or no), assuming that individuals with stable unions are less exposed to roads in their leisure time [32]; and (vi) current drinker, defined as a person who consumes alcohol once or more per month (yes or no), binge drinking, defined as a men who consumed five or more units of alcohol (four or more units for women) on a single occasion in the past 30 days (yes or no), and reports of driving after consuming alcohol (yes or no) [33, 34]. These variables are the main behavioral risk factors for injuries and deaths caused by road traffic crashes [17, 24, 35]. Other behavioral factors such as the use of a helmet and seat belts were not assessed in this study because these variables are mainly related to the severity of RTIs and not their occurrence. Furthermore, the NHS provides no information on the use of such equipment at the time of the reported crash [23].

In the descriptive analysis, the magnitude and uncertainty of the outcomes were estimated by the prevalence of RTI for each mode of transport and the respective 95 % confidence interval (95 % CI). Bivariate and multivariable logistic regression models were applied to assess crude and adjusted odds ratio and their 95 % CIs, respectively.

Variables that presented a *p*-value of less than 0.20 or those that were identified in the literature as relevant determinants of an RTI [23] were used in multivariable analyses. Multivariable models were fitted for each of the three groups (motorcyclists, car/van occupants, and other road users). Stepwise forward methods were used to adjust for possible confounders and test the interactions. Analyses were performed using SPSS (Ver. 18).

The NHS survey was approved by the National Committee of Ethics in Research (CONEP) in June 2013 (protocol # 328.159).

## Results

Table 1 shows the sociodemographic and behavioral characteristics of the study population. The majority of participants were female, aged 30–59 years, and had intermediate or low education. White people accounted for 48.1 % of participants, and 61.2 % lived with a partner. For alcohol use, 26.5 %, 13.7 %, and 24.3 % of the respondents answered that they were a current drinker, binge drinker, and had driven under the influence of alcohol, respectively.

**Table 1:** Sociodemographic and behavioral characteristics of road traffic injuries according to the National Health Survey. Brazil, 2013

Variable	Total *N* = 60,198		Road traffic injury *N* = 1,840^a^	
	%	95 % CI	%	95 % CI
Sex				
Female	52.9	(52.5; 53.3)	31.1	(29.0; 33.2)
Male	47.1	(46.7; 47.5)	68.9	(66.8; 71.0)
Age (years)				
18–29	26,1	(25,7; 26.4)	43.3	(41.1; 45.6)
30–39	21.6	(21.3; 22.0)	28.6	(26.6; 30.7)
40–59	34,2	(33,9; 34.6)	22.2	(20.4; 24.2)
> = 60	18,1	(17.8; 18.4)	5.8	(4.8; 7.0)
Race/Skin color				
White	48.1	(47.7; 48.5)	41.7	(39.4; 44)
Brown	42.6	(42.2; 43.0)	47.4	(45.1; 49.7)
Black	9.3	(9.1; 9.6)	11.0	(9.5; 12.4)
Education				
College or higher	12.7	(12.5; 13.0)	11.5	(10.1; 13.1)
High school	32.8	(32.4; 33.2)	38.9	(36.7; 41.1)
Elementary school	15.5	(15.2; 15.8)	30.9	(28.8; 33.0)
Lower than elementary school	38.9	(38.5; 39.3)	18.8	(17.0; 20.6)
Married/Partner				
Yes	61.2	(60.8; 61.6)	54.3	(52.0; 56.6)
No	38.8	(38.4; 39.2)	45.7	(43.4; 48.0)
Current drinker				
No	73.5	(73.1; 73.8)	57.2	(55.0; 59.5)
Yes	26.5	(26.2; 26.9)	41.8	(40.5; 45.0)
Binge-drinking				
No	86.3	(86.1; 86.6)	72.9^a^	(70.8; 74.9)
Yes	13.7	(13.4; 13.9)	27.1	(25.1; 29.2)
Drinking and driving				
Yes	24.3	(23.5; 25.1)	36.9	(33.3; 40.5)
No	75.7	(74.9; 76.5)	63.1	(59.5; 66.7)

^**a**^1604 individuals self-reported just one road traffic injury episode in the last 12 months

Overall, 1840 participants (3.1 %) reported at least one RTI in the last 12 months; these respondents were predominantly male (68.9 %), aged 18–29 years (43.3 %), had brown skin color (47.4 %), and low education (49.7 %). Regarding alcohol use, 42.8 % were current drinkers, 27.1 % reported binge drinking, and 36.9 % drove under the influence (Table 1).

RTI prevalence in the Southeast (2.4 %; 95 % CI: 1.9; 2.9), South (2.9 %; 95 % CI: 2.2; 3.6), Central-West (4.4 %; 95 % CI: 3.7; 5,1), Northeast (3.4 %; 95 % CI: 3.0; 3.8), and North (4.8 %; 95 % CI: 4.0; 5.7) regions showed three clear levels: low (Southeast), moderate (South and Northeast), and high (Central-West and North).

**Fig. 1:**
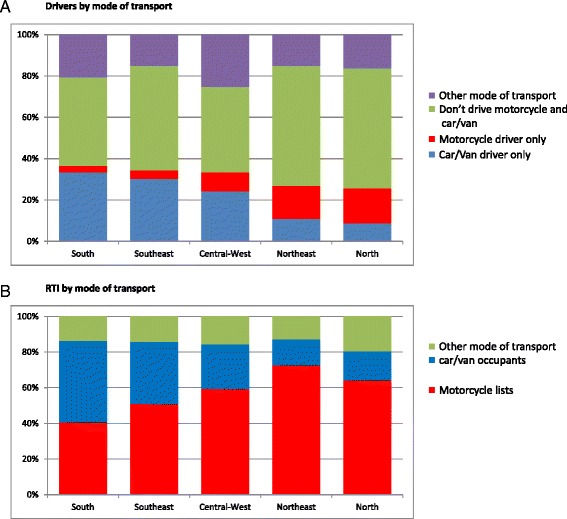
Percentage of (**a**) drivers by mode of transport, 2013 and percentage of (**b**) RTIs by mode of transport and macro-regions of Brazil, 2013.

If we consider only individuals who reported just one RTI in the last 12 months (*N* = 1604), 58.4 % were motorcyclists, 27.1 % were car/van occupants, and 14.4 % traveled by other modes of transport. The distribution of drivers by mode of transport and macro-region is displayed in Fig. 1a. In the North, Northeast and Southeast regions, the proportion of individuals who do not drive cars/vans or motorcycles was greater than that for the other modes of transport. The percentage of car/van drivers was higher in the South and Southeast regions, while higher percentages of motorcyclists drivers were observed in the Northeast and North regions. According to Fig. 1b, motorcyclists were most likely to experience an RTI in all regions, except for the South region where the percentage of car/van occupants was slightly higher than for motorcyclists. The highest percentage of RTIs for motorcyclists was observed in the Northeast and North regions. The Southeast and Central-West regions presented a moderate level of RTIs for car/van occupants and motorcyclists.

Tables 2, 3, and 4 provide results of the overall prevalence of RTIs with respective 95 % CI and adjusted odds ratios according to the mode of transport.

**Table 2:** Prevalence and independent determinants of road traffic injury for motorcycists based on all population. National Health Survey. Brazil 2013

Exposure variables	Total individuals *N* = 60,198	RTI number *N* = 939	RTI prevalence (%)	95 % CI		Adjusted OR	95 % CI		*P* value
				Low	High		Low	High	
Sex									
Female	31638	270	0.85	0.76	0.96				
Male	27895	669	2.40	2.23	2.58	2.63	2.27	3.05	<0.001
Age (years)									
18–29	15447	484	3.13	2.87	3.42	3.20	2.66	3.85	<0.001
30–39	12790	242	1.89	1.67	2.14	2.03	1.67	2.47	<0.001
40–59	20477	186	0.91	0.79	1.05				
> = 60	10819	27	0.25	0.17	0.36	0.30	0.20	0.45	<0.001
Race/Skin color									
White	28237	335	1.19	1.07	1.32				
Brown	25011	486	1.96	1.79	2.13	1.14	0.98	1.33	0.09
Black	5475	109	1.99	1.65	2.40	1.36	1.09	1.70	0.008
Education									
College or higher	7550	80	1.06	0.85	1.32				
High school	19441	342	1.76	1.58	1.95	1.13	0.88	1.46	0.33
Elementary school	9264	220	2.37	2.08	2.71	1.49	1.14	1.94	0.003
Lower than elementary school	23277	296	1.27	1.14	1.42	1.26	0.97	1.63	0.08
Married/Partner									
Yes	36464	506	1.39	1.27	1.51				
No	23069	433	1.88	1.71	2.06	1.07	0.93	1.23	0.35
Binge drinking									
No	51522	704	1.37	1.27	1.47				
Yes	8010	235	2.93	2.59	3.33	1.28	1.10	1.50	0.002
Region									
Southeast	26091	281	1.08	0.96	1.21				
South	8764	90	1.03	0.84	1.26	0.97	0.76	1.24	0.82
Central-West	4370	103	2.36	1.95	2.85	2.01	1.59	2.53	<0.001
Northeast	15890	345	2.17	1.96	2.41	1.78	1.51	2.10	<0.001
North	4418	119	2.69	2.26	3.21	2.02	1.61	2.53	<0.001

**Table 3:** Prevalence and independent determinants of road traffic injury for car/van occupants based on all population. National Health Survey. Brazil 2013

Exposure variables	Total individuals *N* = 60,198	RTI number *N* = 434	RTI prevalence (%)	95 % CI		Adjusted OR	95 % CI		*P* value
				Low	High		Low	High	
Sex									
Female	31531	163	0.52	0.44	0.60				
Male	27497	271	0.99	0.88	1.11	1.74	1.42	2.14	<0.001
Age (years)									
18–29	15090	127	0.84	0.71	1.00	1.50	1.13	2.00	0.01
30–39	12718	170	1.34	1.15	1.55	2.45	1.90	3.16	<0.001
40–59	20389	98	0.36	0.26	0.49				
> = 60	10831	39	0.48	0.39	0.59	1.00	0.68	1.46	0.98
Race/Skin color									
White	28145	243	0.86	0.76	0.98				
Brown	24671	146	0.59	0.50	0.70	0.87	0.70	1.10	0.26
Black	5405	39	0.72	0.53	0.98	1.11	0.78	1.57	0.56
Education									
College or higher	7562	92	1.22	0.99	1.49				
High school	19307	209	1.08	0.95	1.24	0.88	0.68	1.13	0.30
Elementary school	9091	47	0.52	0.39	0.69	0.44	0.31	0.63	<0.001
Lower than elementary school	23066	85	0.37	0.30	0.46	0.40	0.29	0.54	<0.001
Married/Partner									
Yes	36220	262	0.72	0.64	0.82				
No	22808	172	0.75	0.65	0.88	0.99	0.81	1.22	0.94
Binge drinking									
No	51128	310	0.61	0.54	0.68				
Yes	7899	124	1.57	1.32	1.87	2.02	1.62	2.52	<0.001
Region									
Southeast	26001	191	0.73	0.64	0.85				
South	8775	101	1.15	0.95	1.40	1.61	1.26	2.07	<0.001
Central-West	4310	43	1.00	0.74	1.34	1.36	0.97	1.90	0.08
Northeast	15614	69	0.44	0.35	0.56	0.63	0.47	0.84	0.002
North	4329	30	0.69	0.49	0.99	0.99	0.66	1.47	0.95

**Table 4:** Prevalence and independent determinants of road traffic injury for others mode of transport. based on all population. National Health Survey. Brazil 2013

Exposure variables	Total individuals *N* = 60,198	RTI number *N* = 231	RTI prevalence (%)	95 % CI		Adjusted OR	95 % CI		*P* value
				Low	High		Low	High	
Sex									
Female	31368	95	0.30	0.25	0.37				
Male	27226	136	0.50	0.42	0.59	1.42	1.08	1.88	0.01
Age (years)									
18–29	14963	60	0.40	0.31	0.52	0.79	0.55	1.13	0.20
30–39	12548	54	0.43	0.33	0.56	0.99	0.70	1.41	0.96
40–59	20291	85	0.42	0.34	0.52				
> = 60	10792	32	0.30	0.21	0.42	0.65	0.43	0.99	0.046
Race/Skin color									
White	27902	86	0.31	0.25	0.38				
Brown	24524	114	0.46	0.39	0.56	1.20	0.88	1.63	0.24
Black	5366	28	0.52	0.36	0.75	1.41	0.91	2.19	0.13
Education									
College or higher	7470	14	0.19	0.11	0.31				
High school	19098	64	0.34	0.26	0.43	1.59	0.89	2.86	0.12
Elementary school	9044	42	0.46	0.34	0.63	2.13	1.15	3.94	0.02
Lower than elementary school	22982	112	0.49	0.41	0.59	2.53	1.43	4.48	0.001
Married/Partner									
Yes	35957	114	0.32	0.26	0.38				
No	22636	117	0.52	0.43	0.62	1.79	1.36	2.35	<0.001
Binge drinking									
No	50818	175	0.34	0.30	0.40				
Yes	7776	57	0.73	0.57	0.95	1.82	1.33	2.51	<0.001
Region									
Southeast	25810	78	0.30	0.24	0.38				
South	8673	30	0.35	0.24	0.49	1.26	0.82	1.93	0.30
Central-West	4268	27	0.63	0.43	0.92	1.96	1.26	3.06	0.003
Northeast	15545	61	0.39	0.31	0.50	1.09	0.77	1.55	0.61
North	4299	36	0.84	0.61	1.16	2.34	1.54	3.55	<0.001

Table 2 shows that being male, aged 18–29 and 30–39 years, black skin color, having only elementary educational, reporting binge drinking behavior, and living in Central-West, Northeast, or North regions were associated with RTI for motorcyclists. Being 60 years or older was found to be a protective factor against RTIs for motorcyclists (Table 2).

Table 3 shows that being male, aged 18–29 and 30–39 years, reporting binge drinking behavior, and living in the South region were associated with RTI for car/van occupants. The following features were identified as protective factors against RTI for car/van occupants: elementary or lower than elementary-level education, and living in the Northeast region (Table 3).

Finally, Table 4 shows that being male, single, having elementary-level or lower education, reporting binge drinking behavior, and living in the Central-West and North regions were associated with RTI among users of other modes of transport. Being aged 60 years or older was found to be a protective factor (Table 4).

## Discussion

In this study, we found considerable disparities in RTI prevalence among the macro-regions of Brazil. RTI prevalence in the North region was twice as high as in the Southeast region. While the PNAD 2008 [17] showed a high prevalence of RTIs in Central-West and South regions and a low prevalence in the Southeast and North regions, the NHS results showed that the regions with the highest prevalence have now shifted.

Our findings also showed differences in the percentage of RTIs among different modes of transport in each region. In the North and Northeast regions, the percentage of RTIs involving motorcyclists represented more than 60 % of all RTIs, while in the South and Southeast regions the corresponding percentages were 51 % and 41 %, respectively. This investigation showed that the prevalence of RTI for motorcyclists was two times higher in the North, Northeast, and Central-West regions, when compared with the Southeast region. In the South region, the prevalence of RTI for car/van occupants was 1.6 times than those in the Southeast region.

These findings align with results of the Surveillance System for Violence and Accidents (VIVA) survey, which showed an increase in emergency room visits between 2009 and 2011 among motorcyclists; the percentage of injuries suffered by motorcyclists in the Northeast (64.2 %) was much higher than that in the Southeast region (47.8 %) in the VIVA survey [36]. Furthermore, Brazilian states in the Northeast, North and Central-West regions show a high mortality risk for motorcyclists [37].

The results of this investigation reveal that differences among Brazilian regions appear to have increased between 2008 and 2013 [15, 17], likely because of increased presence of cars and motorcycles in the Northeast and North regions. From 2001–2012, these regions experienced the highest increase in cars in Brazil while the Northeast region accounted for the highest growth in motorcycle numbers among all regions, increasing from 1.8–7.7 motorcycles per 100 inhabitants [38]. The sharp increase in the number of motorcycles is associated with increased purchasing power in the country’s poorest regions. The majority of Brazil’s low-income earners and rural populations are concentrated in the North and Northeast regions, and in the last two decades, there has been an increase in the average income of the lowest socioeconomic strata of the population. This trend, accompanied by a reduction in public transportation spending, contributed to an increase in private transport spending, especially spending towards the acquisition of motorcycles [6]. Furthermore, the percentage of individuals using private transport has surpassed that of public transportation between 2003 and 2013 [12].

This scenario leads to a new pattern of population mobility, characterized by the coexistence of a mix of vehicles and pedestrians sharing the same space on roads not originally designed to accommodate high traffic volume [2]. There have been no investments in infrastructure or road safety to adapt to the new motorized reality [3, 39].

There is evidence that the main effective interventions to reduce RTI are improving vehicle and road infrastructure safety [40]. Furthermore, particularly for low an middle-income countries, the most effective measures combine legislation and enforcement initiatives focused on main risk factors, especially excessive speed and alcohol use [41].

Our study pointed to a greater risk of RTI among male occupants in all modes of transport. This finding corroborates reports from the Ministry of Health (2009) [36] and the 2008 PNAD [17]. Results from other developing countries also showed highest RTIs among males [19, 42–44]. However, there is no consensus in the international literature regarding the degree to which RTI differ between males and females [45]. In Spain the risk for males was higher for younger vs. older age groups, and the male/female ratio increased with the severity of the injury. For females, the risk was higher for adults and older adults vs. youth, regardless of the mode of transport and severity of the injury. [45]. In the United States, the risk of non-fatal injuries was higher among males vs. females for pedestrians, cyclists, and vehicle and bus occupants; for motorcyclists, there was no difference between males and females [28]. The higher risks facing young men may be related to gender roles associated with high risk behaviors (e.g., speeding and driving under the influence of alcohol) [45, 46].

The present study shows that individuals aged 18–39 years who were motorcyclists or car/van occupants were more likely to suffer an RTI when compared with other age groups; similar findings are also reported by PNAD 2008 [17]. Other studies have documented the high risk of RTI for young adults in developing countries [19, 28, 43, 45]. Being 60 years or older was protective against RTI for motorcyclists and users of other modes of transport, but not for car occupants. Differences in the risk of RTI between young individuals and older adults can be explained by a greater exposure to traffic by young males, who use motorcycles for urban mobility and who more often exhibit the risky behaviors described above [45].

Among motorcyclists, those who described themselves as black had a greater risk of RTI compared with white participants. For car/van occupants and other road users, no association was found between RTI and skin color. A study conducted in Brazil in 2008 showed that brown and black individuals were six times more likely to die from an RTI than their white counterparts [30]. National and international reports show similar results, with black people exposed to a higher risk of death from traffic crashes, and dying at a younger age from RTIs than white people [30]. The greater risk of RTI for black individuals may be related to their socioeconomic level, which typically positions them as vulnerable road users (motorcyclists, cyclists and pedestrians) [30].

In this study, motorcyclists with elementary school-level education had a greater risk of an RTI than those with a high school education or higher. For car/van occupants, having an elementary-level or lower education was a protective factor against RTIs. For the category “other modes of transport”, individuals with an elementary-level or lower education presented a greater risk of an RTI than those with a college education or higher.

A possible explanation for these findings is that schooling might act as a proxy for socioeconomic level. Individuals from low socioeconomic backgrounds (elementary education or less) that cannot afford a car are more likely to use a motorcycle. Evidence to support this claim can be found in the profile of motorcyclists who suffered an RTI in Brazil; in general they are young males, with elementary school-level education and use a motorcycle daily as their mode of transport [47, 48].

Regarding alcohol consumption, there was a greater risk of RTI among binge drinkers for all modes of transport; for car/van occupants, the magnitude of risk was the highest. A study using data from the NHS to evaluate alcohol consumption showed that residents in the Northeast and Central-West regions of Brazil have the highest levels of binge drinking and driving under the influence [49]. Another study that analyzed NHS data to determine alcohol consumption showed a higher prevalence of binge drinking in the North, Northeast, and Central-West regions compared with the South and Southeast regions of Brazil [50]. The first survey to investigate the profile of alcohol use in Brazil found a high prevalence of driving under the influence with the following risk factors: being male, binge drinking, previous traffic crash while driving under the influence, and an unfavorable opinion score regarding interventions to control drinking and driving [34].

This study identified high RTI prevalence among young males who reported a motorcycle as their main mode of transport; thus, enforcement interventions that focus on reducing speeding and driving under the influence by motorcyclists, especially young males, should be prioritized especially in municipalities located in the North, Northeast and Central-West regions of the country. Additionally, training programs for beginner motorcyclists and measures to improve the visibility of motorcycles should be implemented [24].

The potential limitations of this study should be acknowledged. It would be desirable to estimate the rates of vehicle-specific RTIs by looking at the individuals exposed to each mode of transport using vehicle miles traveled, number of trips, and time spent as a passenger or driver of each mode of transport. However, the NHS survey was not designed to sample sufficient numbers of such exposures. Therefore, some observed disparities may be a result of this differential exposure. Another limitation is the likely presence of recall bias, which could influence the quality of the data collected during the household interview. A further issue is survival bias, which is a common limitation of prevalence studies. In addition, it would be desirable to have a larger number of variables potentially associated with RTIs, such as car and motorcycle ownership, occupation, time spent in urban mobility for each mode of transport, among others.

Despite the limitations of the NHS data in investigating RTI risk factors, our study represents the latest description of RTIs patterns in Brazil, and could be used to guide further investigations, potentially focusing on the regions presenting the highest concentration of RTIs.

## Conclusions

This study identified important regional differences in RTIs. Compared with the 2008 PNAD, our findings showed changes in the magnitude of RTIs and their distribution among regions, reflecting the recent growth in the rates of the motorization of cars/vans and motorcycles in Brazil. This study also identified differences in the magnitude and statistical significance of RTI determinants among motorcyclists, car/van occupants, and other modes of transport.

The results of this investigation can guide interventions to reduce the burden of RTI and reduce differences among regions and modes of transport. To achieve this aim as well as goals set by international agencies -- the goals of the Decade of Action for Road Safety [51] and the Sustainable Development Goals of the United Nations [52] --, Brazil needs to strengthen its transportation and road safety policies by implementing measures such as: (i) creation of a lead agency for the national management of road safety with the power and ability to formulate and conduct a national road safety action plan and coordinate inter-sectoral initiatives; (ii) implementation of a national information system for RTI to unify data on road traffic crashes for the three levels of government (federal, state and municipal), and enable the collection of police and health sector data on injured individuals; (iii) creation of a systematic and regular survey for data collection concerning the urban mobility of the population for each mode of transport to allow the estimation of RTI rates based on exposure to various modes of transport; (iv) expansion and improvement of the quality of public transport supply in urban areas and reduction of the subsidy for the production of cars and motorcycles; (v) implementation of safety infrastructure for urban roads and highways to reduce the risk of road traffic crashes and to enable a peaceful coexistence between the various modes of transport; and finally, (vi) review the National Traffic Code regarding the main risk factors (e.g., excessive speed, driving under the influence, and inappropriate behavior by motorcyclists). Important changes to the code should include, banning of motorcycle traffic between the two lanes of urban highways and roads, tightening the requirements for motorcyclists to obtain a driver’s license, and creating regulation aimed at improving motorcyclists’ conspicuity. (vii) strengthening the enforcement and punishment to reduce driving under influence of alcohol and excessive speed.

Inter-sectoral structural measures led by the Federal Government together with the states and municipalities may help to reduce the magnitude of RTI in Brazil and address regional inequalities in the distribution of RTIs. Furthermore, targeted road safety interventions for vulnerable road users such as pedestrians and motorcyclists, and the population groups most likely to experience RTI, as identified in this study, is vital to reduce inequalities within the population.

## Abbreviations

BRICS: Brazil, Russia, India, China, and South Africa
CI: Confidence interval
CONEP: National committee of ethics in research
IBGE: Brazilian institute of geography and statistics
MoH: Ministry of health
NHS: National health survey
OR: Odds ratio
PNAD: National survey by household sample
RTI: Road traffic injury
SIH: Hospital information system
VIVA: Violence and accidents surveillance system

## References

[1] Global Road Safety Facility, The World Bank, Institute for Health Metrics and Evaluation. Transport for health: The global Burden of Disease from Motorized Road Transport. [Internet]. Seattle, WA: IHME ; Washington, DC: The World Bank; 2014. Available from: http://documents.worldbank.org/curated/pt/984261468327002120/pdf/863040IHME0T4H0ORLD0BANK0compressed.pdf

[2] Hyder AA, Vecino-Ortiz AI (2014). BRICS: opportunities to improve road safety. Bull World Health Organ.

[3] Vasconcellos EDA. Risco no trânsito, omissão e calamidade: Impactos do incentivo à motocicleta no Brasil. 2013;90. Available from: http://files-server.antp.org.br/_5dotSystem/download/dcmDocument/2013/08/29/0D2E1C9E-38D9-478A-A24D-BB121A3A295A.pdf

[4] Marim D. Estratégias na indústria de motocicletas: Um estudo exploratório do setor de motocicletas brasileiro. Pontifícia Universidade Católica de São Paulo; 2010. http://livros01.livrosgratis.com.br/cp146220.pdf.

[5] IPEA (2013). Indicadores de mobilidade urbana da PNAD 2012. Comun do IPEA.

[6] IPEA. Gastos das famílias brasileiras com transporte urbano público e privado no Brasil: Uma análise da POF 2003 e 2009. Brasília; 2012. http://www.ipea.gov.br/portal/images/stories/PDFs/TDs/td_1803.pdf.

[7] Morais Neto OL, Montenegro M de MS, Monteiro RA, Rodrigues FR, Botacin CF, Beniz LAF. Road Traffic Injuries - Profile and Trends - Brasil, 2004–2013. Heal. Brazil 2014 a situational Anal. Road Inj. other Extern. causes [Internet]. Editor. Brasilia: Ministry of Health of Brazil Publisher; 2015. p. 117–44. Available from: http://bvsms.saude.gov.br/bvs/publicacoes/health_brazil_2014_situational_analysis.pdf

[8] Morais Neto OL, Montenegro MDMS, Monteiro RA, Siqueira Júnior JB, Silva MMA, Lima CM (2012). Mortalidade por acidentes de transporte terrestre no Brasil na última década: tendência e aglomerados de risco. Cien Saude Colet.

[9] IPEA. Desafios da mobilidade urbana no Brasil. Brasilia; 2016. http://www.ipea.gov.br/portal/images/stories/PDFs/TDs/td_1803.pdf.

[10] Szwarcwald CL, Damacena GN, Souza Júnior PRB, Almeida WDS, Malta DC (2016). Percepção da população brasileira sobre a assistência prestada pelo médico. Brasil, 2013. Cien Saude Colet.

[11] Liberatti C, Andrade S, Soares D (2001). The new Brazilian traffic code and some characteristics of victims in southern Brazil. Inj Prev.

[12] ANTP. Associação Nacional de Transportes Públicos. Sistema de Informações da Mobilidade Urbana Relatório Comparativo 2003–2013 [Internet]. Assoc Nac Transp Públicos. 2015 [cited 2015 Aug 15]. 118. Available from: http://files-server.antp.org.br/_5dotSystem/userFiles/SIMOB/Relatorio%20Comparativo%202013.pdf.

[13] Brasil, Ministério da Saúde, Secretaria de Vigilância em Saúde. Departamento de Vigilância de Doenças e Agravos não Transmissíveis e Promoção da Saúde. Sistema de Vigilância de Violências e Acidentes (VIVA) : 2009, 2010 e 2011 [Internet]. 2013 [cited 2016 Jan 1]. Available from: http://bvsms.saude.gov.br/bvs/publicacoes/sistema_vigilancia_violencia_acidentes.pdf.

[14] Andreuccetti G, Carvalho H, Cherpitel C, Yu Y, Ponce J, Kahn T (2011). Reducing the legal blood alcohol concentration limit for driving in developing countries: a time for change? Results and implications derived from a time series analysis (2001–2010) conducted in Brazil: reducing blood alcohol limits for driving. NHI Public Access.

[15] Instituto Brasileiro de Geografia e Estatística I (2010). Um panorama da saúde no Brasil: Acesso e utilização dos serviços, condições de saúde e fatores de risco e proteção à saúde.

[16] Instituto Brasileiro de Geografia e Estatística I (2015). Pesquisa nacional de saúde: 2013 : acesso e utilização dos serviços de saúde, acidentes e violências : Brasil, grandes regiões e unidades da federação.

[17] Malta DC, Mascarenhas MDM, Bernal RTI, da Silva MMA, Pereira CA, Minayo MCDS (2011). Analysis of the occurrence of traffic injuries and related factors according to the National Household Sample Survey (PNAD) Brazil, 2008. Cien Saude Colet.

[18] ÁVila-Burgos L, Medina-Solís CE, Pérerez-Núñez R, Híjar-Medina M, Aracena-Genao B, Hidalgo-Solórzano E, et al. Prevalencia de accidentes de transito no fatales en Mexico: Resultados de la ENSANUT 2006. Salud Publica Mex. 2008;50. 18373007

[19] Gutiérrez JP, Rivera-Dommarco J, Shamah-Levy T, Villalpando-Hernández S, Franco A, Cuevas-Nasu L (2012). Encuesta Nacional de Salud y Nutrición 2012.

[20] WHO. Global status report on road safety. Inj Prev. 2015. Available from: http://apps.who.int/iris/bitstream/10665/42871/1/9241562609.pdf 10.1136/ip.2009.023697 19652008

[21] Berecki-Gisolf J, Yiengprugsawan V, Kelly M, McClure R, Seubsman SA, Sleigh A (2015). The impact of the thai motorcycle transition on road traffic injury: Thai cohort study results. PLoS One.

[22] Dandona R, Kumar GA, Ameer A, Ahmed GM (2008). Underreporting of road trafic injuries to the police: results from two data soureces in urban India. Inj Prev.

[23] WHO. World report on road traffic injury prevention. Geneva; 2004. Available from: http://apps.who.int/iris/bitstream/10665/42871/1/9241562609.pdf

[24] Lin MR, Kraus JF (2009). A review of risk factors and patterns of motorcycle injuries. Accid Anal Prev.

[25] Bacchieri G, Barros AJD (2011). Acidentes de trânsito no Brasil de 1998 a 2010: Muitas mudanças e poucos resultados. Rev Saude Publica.

[26] Instituto Brasileiro de Geografia e Estatística I. Pesquisa Nacional de Saúde - 2013: percepção do estado de saude, estilos de vida e doenças crônicas - Brasil, Grandes Regiões e Unidades da Federação. Rio de Janeiro; 2014.

[27] Malta DC, Santos MAS, Stopa SR, Vieira JEB, Melo EA, Reis AAC. A Cobertura da Estratégia de Saúde da Família (ESF) no Brasil, segundo a Pesquisa Nacional de Saúde, 2013 Family Health Strategy Coverage in Brazil, according to the National Health Survey, 2013. Cien Saude Colet. 2013;327–38. 10.1590/1413-81232015212.23602015 26910142

[28] Beck LF, Dellinger AM, O’Neil ME (2007). Motor vehicle crash injury rates by mode of travel, United States: Using exposure-based methods to quantify differences. Am J Epidemiol.

[29] Marín L, Queiroz MS. Present status of traffic accidents in the age of speed: an overview. Cad. saude publica / Minist. da Saude, Fund. Oswaldo Cruz, Esc. Nac. Saude Publica. 2000;16:7–21. 10.1590/s0102-311x2000000100002 10738146

[30] Araujo EM, Costa MDCN, Hogan VK, Mota ELA, Araujo TM, Oliveira NF (2009). Diferenciais de raca/cor da pele em anos potenciais de vida perdidos por causas externas. Rev Saude Publica.

[31] Almeida GCM de, Medeiros F da CD, Pinto LO, Moura JMB de O, Lima KC. Prevalência e Fatores Associados a Quedas em Idosos. Texto Context. Enferm. 2016;25:435–42. Available from: http://www.scielo.br/pdf/tce/v25n2/0104-0707-tce-25-02-0360015.pdf.

[32] Abreu ÂMM, De Lima JMB, Matos LN, Pillon SC (2010). Uso de álcool em vítimas de acidentes de trânsito: estudo do nível de alcoolemia. Rev Lat Am Enfermagem.

[33] Brasil. Secretaria de Vigilância em Saúde. Departamento de Vigilância de Doenças e Agravos não transmissíveis e Promoção da Saúde. Vigitel Brasil 2014: vigilância de fatores de risco e proteção para doenças crônicas por inquérito telefônico / Ministério da Saúde, Secretaria deVigilância em Saúde, Departamento de Vigilância de Doenças e Agravos não Transmissíveis e Promoção da Saúde. Brasília: Ministério da Saúde. 2015;152l. ISBN 978-85-334-2243-8.

[34] Pechansky F, De Boni R, Von Diemen L, Bumaguin D, Pinsky I, Zaleski M (2009). Highly reported prevalence of drinking and driving in Brazil: Data from the first representative household study. Rev Bras Psiquiatr.

[35] Lim SS, Vos T, Flaxman AD, Danaei G, Shibuya K, Adair-Rohani H (2012). A comparative risk assessment of burden of disease and injury attributable to 67 risk factors and risk factor clusters in 21 regions, 1990–2010: A systematic analysis for the Global Burden of Disease Study 2010. Lancet.

[36] Andrade SSC de A, Sá NNB de, Carvalho MGO de, Lima CM, Silva MMA da, Neto OLM, et al. Perfil das vítimas de violências e acidentes atendidas em serviços de urgência e emergência selecionados em capitais brasileiras: Vigilância de Violências e Acidentes, 2009. Rev. Epidemiol. e Serviços Saúde. 2012;21:21–30.

[37] Martins ET, Boing AF, Peres MA (2013). Motorcycle accident mortality time trends in Brazil, 1996–2009. Rev Saude Publica.

[38] Observatório das Metrópoles (2013). Evolução da Frota de Automóveis E Motos No Brasil 2001 – 2012 (Relatório 2013). Inst Nac Ciencias e Tecnol.

[39] Hyder AA, Bishai D (2012). Road Safety in 10 Countries: A Global Opportunity. Traffic Inj Prev.

[40] Novoa AM, Pérez K, Borrell C (2009). Efectividad de las intervenciones de seguridad vial basadas en la evidencia: una revisión de la literatura. Gac Sanit.

[41] Staton C, Vissoci J, Gong E, Toomey N, Wafula R, Abdelgadir J (2016). Road Traffic Injury Prevention Initiatives: A Systematic Review and Metasummary of Effectiveness in Low and Middle Income Countries. PLoS One.

[42] Dibben C, Popham F (2013). Are health inequalities evident at all ages? An ecological study of English mortality records. Eur J Public Health.

[43] Dandona R, Kumar GA, Raj TS, Dandona L (2006). Patterns of road traffic injuries in a vulnerable population in Hyderabad, India. Inj Prev.

[44] Keng SH (2005). Helmet use and motorcycle fatalities in Taiwan. Accid Anal Prev.

[45] Santamariña-Rubio E, Pérez K, Olabarria M, Novoa AM (2014). Gender differences in road traffic injury rate using time travelled as a measure of exposure. Accid Anal Prev.

[46] Courtenay WH (2000). Constructions of masculinity and their influence on men’s well-being: A theory of gender and health. Soc Sci Med.

[47] Factor R, Mahalel D, Yair G (2008). Inter-group differences in road-traffic crash involvement. Accid Anal Prev.

[48] Híjar M, Arredondo A, Carrillo C, Solórzano L (2004). Road traffic injuries in an urban area in Mexico: An epidemiological and cost analysis. Accid Anal Prev.

[49] Macinko J, Mullachery P, Silver D, Jimenez G, Neto OLM (2015). Patterns of alcohol consumption and related behaviors in Brazil: Evidence from the 2013 National Health Survey (PNS 2013). PLoS One.

[50] Garcia LP, Freitas LRS De. Consumo abusivo de álcool no Brasil: resultados da Pesquisa Nacional de Saúde 2013. Epidemiol. e Serviços Saúde [Internet]. 2015;24:227–37. Available from: http://www.scielo.br/pdf/ress/v24n2/2237-9622-ress-24-02-00227.pdf

[51] United Nations. Resolution adopted by the General Assembly-64/255. Improving global road safety [Internet]. RES A/64/255. 2010 [cited 2015 Aug 27]. p. 1–6. Available from: http://www.who.int/violence_injury_prevention/publications/road_traffic/UN_GA_resolution-54-255-en.pdf

[52] United Nations. Transforming our world: The 2030 agenda for sustainable development. A/RES/70/1. https://sustainabledevelopment.un.org/content/documents/21252030%20Agenda%20for%20Sustainable%20Development%20web.pdf. 2015;1–5.

